# Effects of cold exposure revealed by global transcriptomic analysis in ferret peripheral blood mononuclear cells

**DOI:** 10.1038/s41598-019-56354-6

**Published:** 2019-12-27

**Authors:** Bàrbara Reynés, Evert M. van Schothorst, Jaap Keijer, Andreu Palou, Paula Oliver

**Affiliations:** 1 Laboratory of Molecular Biology, Nutrition and Biotechnology (Nutrigenomics and Obesity group), University of the Balearic Islands, Palma, Spain; 2 Health Research Institute of the Balearic Islands (IdISBa), Palma, Spain; 3 CIBER de Fisiopatología de la Obesidad y Nutrición (CIBEROBN), Madrid, Spain; 4 Human and Animal Physiology, Wageningen University, Wageningen, The Netherlands

**Keywords:** RNA, Transcriptomics

## Abstract

Animal studies, mostly performed in rodents, show the beneficial anti-obesity effects of cold studies. This is due to thermogenic activation of brown adipose tissue (BAT), a tissue also recently discovered in adult humans. Studies in humans, however, are hampered by the accessibility of most tissues. In contrast, peripheral blood mononuclear cells (PBMC) are accessible and share the expression profile of different sets of genes with other tissues, including those that reflect metabolic responses. Ferrets are an animal model physiologically closer to humans than rodents. Here, we investigated the effects on ferrets of one-week acclimation to 4 °C by analysing the PBMC transcriptome. Cold exposure deeply affected PBMC gene expression, producing a widespread down-regulation of genes involved in different biological pathways (cell cycle, gene expression regulation/protein synthesis, immune response, signal transduction, and genes related to extracellular matrix/cytoskeleton), while thermogenic and glycogenolysis-related processes were increased. Results obtained in PBMC reflected those of adipose tissue, but hardly those of the liver. Our study, using ferret as a model, reinforce PBMC usefulness as sentinel biological material for cold-exposure studies in order to deepen our understanding of the general and specific pathways affected by cold acclimation. This is relevant for future development of therapies to be used clinically.

## Introduction

The effects of cold exposure on mammals have mainly been studied in terms of its relevance for body temperature and body weight maintenance. It is well established that sympathetic nervous system activation in brown adipose tissue (BAT) by cold exposure increases energy expenditure and induces adaptive thermogenesis in order to preserve body temperature at the expense of mobilisation of fat stores, which helps to maintain body weight^1,2^. More recently, BAT-thermogenic activation capacity by cold exposure has also been described in humans, which opens new possibilities for protection against obesity^3^. In addition, cold acclimation has been widely studied in relation to modulation of the inflammatory response, and although some research points to an immunosuppressive effect, there are contradictory results^4,5^. Furthermore, the modulatory effect of environmental temperature is more complex, and has an important regulatory role in overall cellular processes^6,7^. Mechanistic research on the effects of cold exposure mostly examine gene expression responses of selected specific tissues in animal models, mainly rodents, or cell lines^6,8^, which makes it difficult to gain a broad idea of the whole metabolic effect of cold on our metabolism. In general, these studies show that low temperature stress induces a slowing in a range of cellular processes, but potentiates pathways involved in heat production^9^.

Peripheral blood mononuclear cells (PBMC), a fraction of blood cells composed of lymphocytes and monocytes, constitute an important source of biomarkers of biological effects in internal tissues^10^. Studies by our group performed in rodents reveal that PBMC are able to reflect BAT thermogenic activation as well as the acquisition of brown-like properties in white adipose tissue (browning process) in response to cold exposure, pointing towards the possibility of performing thermogenic studies with minimum invasiveness^11^. In general, these circulating blood cells express most of the genes encoded by the genome (including tissue-specific transcripts), and can respond to internal and external signals reflecting gene expression patterns of several tissues (reviewed by^10,12^). So, in addition to revealing the thermogenic adaptations to cold exposure, PBMC reflect gene expression changes in response to nutritional and pharmacological interventions, and are increasingly used in nutrigenomic and clinical research as sentinel biological material to study metabolic responses of other internal tissues that are difficult to obtain^10^. In this sense, we have demonstrated, in rats and ferrets, that PBMC can even be useful to study hypothalamic responses to cold exposure^13^. Ferrets (*Mustela putorius furo*) are an alternative animal model to rodents that are closer to humans in various aspects, including adipose tissue organisation and thermogenic and immune response to cold exposure^14–17^. Recently, we showed in ferrets that an immunosuppressive gene expression pattern was specifically induced by cold acclimation in perivascular adipose tissue but not in inguinal adipose tissue^18^. A limited set of inflammation markers was also analysed in PBMC and reflected the perivascular adipose tissue response^18^. Given the deleterious impact of inflammation on vascular biology, this could be indicative of a cardiovascular protective effect^18^.

PBMC, given their particular characteristics, constitute an ideal surrogate tissue to study the global biological and molecular responses to cold exposure in mammals. Taking into consideration that PBMC are immune cells, and since inflammation is the basis of several metabolic diseases – such as obesity or cardiovascular disease^19^ – detailed analysis of PBMC gene expression analysis could serve to better understand immune modulation by cold. Therefore, here we performed a global transcriptome gene expression study (microarray analysis) of PBMC in control and cold-exposed ferrets to characterise the effect of cold exposure on metabolism. The most outstanding gene expression patterns were reflected in a homeostatic tissue with a key role in cold response – adipose tissue – but hardly in the liver.

## Results

### Body weight, adiposity, and serum parameters

Data of body weight, adiposity, and circulating NEFA levels of the same set of animals have previously been published^18^. Briefly, cold exposure induced a reduction in fat mass, with a significant decreased size of both subcutaneous (inguinal and interscapular) and visceral (retroperitoneal) adipose tissue depots in cold-exposed animals. Despite the lower fat mass, final body weight was not affected, although animals in the cold group had a lower body weight increase (−16%) in comparison to controls during the week in which they were exposed to cold. As expected, and in agreement with the mobilisation of fat depots, circulating NEFA levels were increased by 35% in the cold group. Circulating glucose levels were not affected by cold acclimation (187 ± 21 mg/dL for cold *vs* 127 ± 17 mg/dL for control groups; p = 0.06 by Students’ *t*-test).

### PBMC transcriptomic analysis

**Figure 1:**
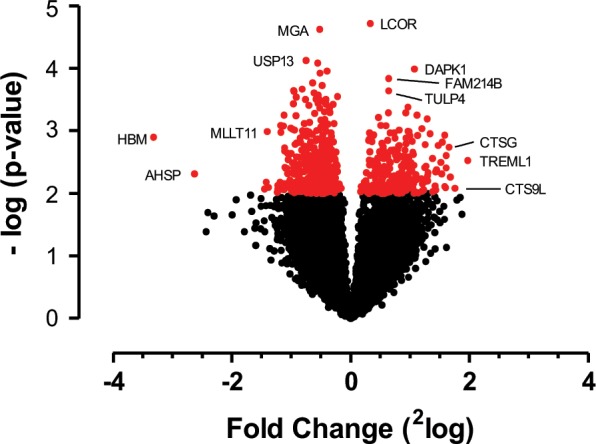
Volcano plot of unique genes expressed in PBMC plotted as 2 log fold change of cold over control exposed ferrets versus the P-value. Non-significant genes are shown in black, significant genes (p < 0.01; Top 50 shown in Table 2) in red. Gene symbols are shown for a subset of significant genes on the perimeter.

In order to gain a global, comprehensive overview of the impact of cold exposure on global gene expression, we developed a ferret microarray consisting of 45,328 sequences, encoding a total of 19,299 unique genes. We then performed a global gene expression analysis of PBMC of ferrets acclimatised to 4 °C (cold) or 22 °C (control) for one week. Our results show that PBMC gene expression was affected by cold exposure with a total of 611 genes changing their expression in cold *vs* control animals (considering a p-value < 0.01), 61% of which were down-regulated. A volcano plot of these genes is shown in Fig. 1, and the heatmap in Supplementary Fig. 1.

**Figure 2:**
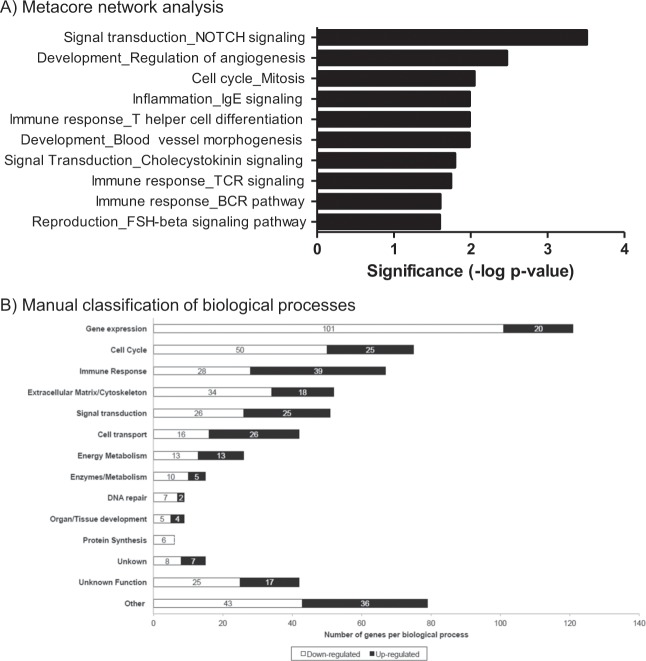
Classification of genes affected by cold exposure (Students’ *t*-test, p < 0.01). (**A**) Network enrichment analysis using Metacore. (**B**) Manual classification of genes into biological process.

For interpretation, a bioinformatics network analysis was performed (Fig. 2A), which identified immune response as being highly differentially expressed. Within the top10 networks, five were immune related, with three additional ones being related to blood vessel morphogenesis/angiogenesis/signalling. For a more specific view, the 611 top regulated genes were manually classified in biological processes, using scientific literature and databases. Each individual gene was included in one process only. This classification resulted in 11 processes: gene expression, cell cycle and immune response, extracellular matrix or cytoskeleton, signal transduction, cell transport, energy metabolism, enzymes/metabolism, DNA repair, organ and tissue development and protein synthesis (Fig. 2B). The other categories were unknown genes (n = 15), genes with an unknown function (n = 42), and genes involved in several different processes (n = 79).

For a more detailed analysis, the three most regulated processes were sub-classified into different sub-processes (Table 1). Genes involved in gene expression were sub-classified into transcription factors, RNA maturation, DNA structure, epigenetic regulation, co-activators and co-repressors, post-transcriptional regulators, interfering RNA (siRNA, miRNA), RNA degradation, and others, with transcription factors as the most abundant sub-classification; all of which were mainly down-regulated by cold exposure. Regarding cell cycle and fate, cold exposure especially regulated genes involved in cell proliferation, cell cycle regulation, apoptosis, and cell differentiation and determination; these genes were mainly down-regulated, especially those involved in cell proliferation. Finally, the third most regulated pathway involved immune response-related genes, which were sub-classified into immune system maturation and activation, antigen recognition and presentation, antigen degradation, cytokine signalling, and others: 28 of these genes were down-regulated, while 39 were up-regulated (Table 1 and heatmap shown in Supplementary Fig. 2). The top 50 regulated genes affected by cold exposure based on their p-value are listed in Table 2, and the associated heatmap is shown in Supplementary Fig. 3. Coincident with results of the general pathway analysis shown in Fig. 2, most of the top regulated genes were involved in gene expression, mainly for transcription factors, which were down-regulated. The regulated genes involved in energy metabolism were also studied in more detail and sub-classified according to their function in carbohydrate metabolism, lipid metabolism, energy balance regulation, respiratory chain and mitochondrial ATPase system, thermogenesis and others (Table 3). The relevance and implications of all the changes observed in the transcriptome analysis are described in detail in the Discussion section.

**Table 1:** Most relevant affected pathway by cold exposure.

Classification	Sub-classification	Number of genes	
		Up-regulated	Down-regulated
**Gene expression**			
	Transcription factor	13	53
	RNA maturation	2	10
	DNA structure	2	9
	Epigenetic regulation	0	6
	Coactivator	0	5
	Post-transcriptional regulation	0	5
	Corepressor	0	4
	Interfering RNA (siRNA, miRNA)	0	2
	RNA degradation	1	0
	Others	2	7
**Cell cycle**			
	Cell proliferation	8	21
	Cell cycle regulation	9	16
	Apoptosis	3	6
	Cell differentiation	3	6
	Cell determination	1	2
**Immune response**			
	Immune system maturation/activation	26	17
	Antigen recognition/presentation	5	5
	Antigen degradation	3	2
	Cytokine signalling	2	1
	Others	3	3

Sub-classification of most relevant affected pathway by cold exposure in PBMC based on the top regulated genes (Students’ *t*-test, p < 0.01).

**Table 2:** Classification of top 50 regulated genes affected by cold exposure.

Gene Name	Gene Symbol	Sequence ID	Fold Change	p-value
**Gene expression**				
Zinc Finger BED-Type Containing 5	*Zbed5*	XM_004751989.2	−2.18	5.58E-04
Trinucleotide Repeat Containing 6A	*Tnrc6a*	XM_013048889.1	−1.76	2.15E-04
Lysine acetyltransferase 6B	*Kat6b*	XM_004769363.2	−1.70	3.14E-04
Zinc finger CCHC-type containing 11	*Zcchc11*	XM_013046839.1	−1.62	7.24E-04
Exportin For TRNA	*Xpot*	XM_004752498.2	−1.47	8.22E-05
Zinc Finger Protein 770	*Znf770*	XM_004751051.2	−1.46	3.59E-04
MGA, MAX Dimerization Protein	*Mga*	XM_004751157.2	−1.44	2.37E-05
Zinc Finger MYM-Type Containing 5	*Zmym5*	XM_013048828.1	−1.39	1.87E-04
Zinc Finger And SCAN Domain Containing 30	*Zscan30*	XM_004752298.2	−1.37	3.35E-04
Enhancer Of Polycomb Homolog 1	*Epc1*	XM_004777872.2	−1.36	4.94E-04
Zinc Finger Protein 235	*Znf235*	XM_013063895.1	−1.22	7.59E-04
Tubby Like Protein 4	*Tulp4*	XM_013044950.1	1.56	2.27E-04
**Cell cycle**				
Family with sequence similarity 65 member B	*Fam65b*	XM_004761237.2	−1.72	7.54E-04
Protein tyrosine phosphatase type IVA, member 3	*Ptp4a3*	XM_004743631.2	−1.54	2.45E-04
MDM4, p53 regulator	*Mdm4*	XM_004756399.2	−1.42	6.66E-04
Ubiquitin Specific Peptidase 37	*Usp37*	XM_004762758.2	−1.33	3.70E-04
Fibronectin type III domain containing 3B	*Fndc3b*	XM_004756587.2	1.38	6.09E-04
Structural Maintenance Of Chromosomes 1B	*Smc1b*	XM_004771114.2	1.95	4.15E-04
Death associated protein kinase 1	*Dapk1*	XM_004774132.2	2.10	1.02E-04
**Extracellular matrix/Cytoskeleton**				
Septin 1	*Sept1*	XM_004741975.2	−1.95	3.66E-04
BBSome Interacting Protein 1	*Bbip1*	XM_004763864.1	−1.95	2.24E-04
Coiled-Coil Serine Rich Protein 2	*Ccser2*	XM_004765771.2	−1.50	3.08E-04
Zinc Finger RANBP2-Type Containing 1	*Zranb1*	XM_004751744.2	−1.33	3.13E-04
Syntaxin Binding Protein 6	*Stxbp6*	XM_004755163.2	−1.23	3.72E-04
**Immune response**				
C-type lectin domain family 2 member D	*Clec2d*	XM_004779708.2	−2.27	8.13E-04
Tripartite Motif Containing 13	*Trim13*	XM_004775233.2	−1.43	1.19E-04
Sialic Acid Binding Ig Like Lectin 8	*Loc101680639*	XM_004767130.2	2.44	6.39E-04
Human immunodeficiency virus type I enhancer binding protein 2	*Hivep2*	XM_013056235.1	−1.89	2.89E-04
**Cell transport**				
TBC1 Domain Family Member 16	*Tbc1d16*	XM_004748756.2	−1.45	3.09E-04
Stromal Interaction Molecule 2	*Stim2*	XM_004762647.2	−1.35	6.52E-04
**Energy metabolism**				
UDP-GlcNAc:BetaGal Beta-1,3-N-Acetylglucosaminyltransferase 5	*B3gnt5*	XM_004745085.2	−1.90	5.68E-04
Nucleotide Binding Protein Like	*Nubpl*	XM_013047446.1	−1.17	2.81E-04
**Signal transduction**				
Mitogen-Activated Protein Kinase Kinase 6	*Map2k6*	XM_004749075.2	−1.56	1.70E-04
Dmx Like 2	*Dmxl2*	XM_004755431.2	2.17	5.59E-04
Enzymes/metabolism				
Cysteine Sulfinic Acid Decarboxylase	*Csad*	XM_004774642.1	−1.46	2.66E-04
**Other**				
Prostate transmembrane protein, androgen induced 1	*Pmepa1*	XM_004759915.2	−2.26	8.15E-04
Ubiquitin Specific Peptidase 13 (Isopeptidase T-3)	*Usp13*	XM_004756639.1	−1.68	7.44E-05
Low Density Lipoprotein Receptor Class A Domain Containing 4	*Ldlrad4*	XM_013051371.1	−1.63	3.90E-04
SUMO1/Sentrin Specific Peptidase 5	*Senp5*	XM_004745326.2	−1.35	8.18E-04
Tau tubulin kinase 2	*Ttbk2*	XM_004751214.1	−1.29	4.40E-04
Atlastin GTPase 2	*Atl2*	XM_004765501.2	−1.27	5.06E-04
Family with sequence similarity 214, member B	*Fam214b*	XM_013053738.1	1.56	1.44E-04
Lysophosphatidylcholine Acyltransferase 2	*Lpcat2*	XM_004744201.2	1.90	5.13E-04
**Unknown function**				
Family With Sequence Similarity 214 Member A	*Fam214a*	XM_004755399.2	−1.94	2.32E-04
Leucine Rich Pentatricopeptide Repeat Containing	*Lrpprc*	XM_013056345.1	−1.53	5.24E-04
Coiled-Coil Domain Containing 181	*Ccdc181*	XM_004756660.2	−1.44	4.31E-04
Protein O-linked mannose N-acetylglucosaminyltransferase 2 (beta 1,4-)	*Pomgnt2*	XM_004754292.2	−1.35	7.68E-04
Transmembrane Protein 19	*Tmem19*	XM_004752428.2	−1.32	1.10E-04
PiggyBac Transposable Element Derived 1	*Pgbd1*	XM_004779982.2	−1.31	7.76E-04
Family With Sequence Similarity 217 Member B	*Fam217b*	XM_004759963.2	1.26	1.92E-05

Detailed classification of top 50 regulated genes affected by cold exposure in PBMC *vs* their respective controls, based on their p-value (Students’ *t*-test). Fold change: + indicates up-regulation, while − indicates down-regulation by cold exposure. Genes are ordered by fold change. This Table is also available as Supplementary data set in .xlsx format.

**Table 3:** Classification of energy metabolism related genes affected by cold exposure.

Gene name	Gene Symbol	Sequence	Fold Change	p-value
**Carbohydrate metabolism**				
Enolase 2 (gamma, neuronal)	*Eno2*	XM_004766643.1	−1.80	8.11E-03
Serine/threonine-protein phosphatase 4 regulatory subunit 3B	*Smek2*	XM_004780298.1	−1.49	9.11E-03
TBC1 Domain Family Member 4	*Tbc1d4*	XM_004754377.1	−1.72	7.45E-03
Glucosidase alpha, acid	*Gaa*	XM_004748753.1	1.42	2.39E-03
Glyceraldehyde-3-Phosphate Dehydrogenase	*Gapdh*	XM_004766702.1	1.60	2.03E-03
Glycogen phosphorylase L	*Pygl*	XM_004738765.1	1.62	6.32E-03
Maltase-glucoamylase	*Mgam*	XM_004741983.1	2.42	3.63E-03
Phosphoglucomutase 2	*Pgm2*	XM_004763981.1	1.81	3.65E-03
Ribose 5-phosphate isomerase A	*Rpia*	XM_004742384.1	1.40	3.96E-03
Solute Carrier Family 2 Member 6	*Slc2a6*	XM_004757400.1	1.48	8.94E-03
**Lipid metabolism**				
Alpha-2-glycoprotein 1, zinc-binding	*Loc101679584*	XM_004781837.1	−1.67	1.97E-03
Enoyl-CoA hydratase and 3-hydroxyacyl CoA dehydrogenase	*Ehhadh*	XM_004745171.1	−1.45	3.01E-03
Lipin 1	*Lpin1*	XM_004745812.1	−1.51	2.58E-03
Low Density Lipoprotein Receptor Adaptor Protein 1	*Ldlrap1*	XM_004741138.1	−2.18	8.69E-03
Protein Inhibitor Of Activated STAT 1	*Pias1*	XM_004758612.1	−1.44	5.26E-03
Scavenger receptor class F member 1	*Scarf1*	XM_004746870.1	−1.46	4.62E-03
Emopamil binding protein-like	*Ebpl*	XM_004775264.1	1.97	2.76E-03
Transmembrane 6 Superfamily Member 1	*Tm6sf1*	XM_004763661.1	2.00	6.15E-03
**Energy balance regulators**				
Pyruvate Dehydrogenase Kinase 1	*Pdk1*	XM_004744080.1	−2.16	2.08E-03
Sirtuin 1	*Sirt1*	XM_004777825.1	−1.21	6.05E-03
**Respiratory chain/mitochondrial ATPase system**				
Nucleotide Binding Protein Like	*Nubpl*	XM_004775760.1	−1.17	2.81E-04
NDUFA4, mitochondrial complex associated	*Loc101671166*	XM_004743436.1	1.26	5.09E-03
**Thermogenesis**				
Peptidase M20 Domain Containing 1	*Pm20d1*	XM_004756447.1	1.93	6.18E-03
Transmembrane protein 56	*Tmem56*	XM_004752547.1	1.97	4.87E-03
**Others**				
UDP-GlcNAc:BetaGal Beta-1,3-N-Acetylglucosaminyltransferase 5	*B3gnt5*	XM_004745088.1	−1.90	5.68E-04
Arginase 2	*Arg2*	XM_004738993.1	2.60	2.74E-03

Detailed classification of energy metabolism related genes affected by cold exposure in PBMC *vs* their respective controls, based on their p-value (Students’ *t*-test). Fold change: + indicates up-regulation, while – indicates down-regulation by cold exposure. Genes are ordered by fold change. This Table is also available as Supplementary data set in.xlsx format.

### Array confirmation by real-time RT-qPCR

**Figure 3:**
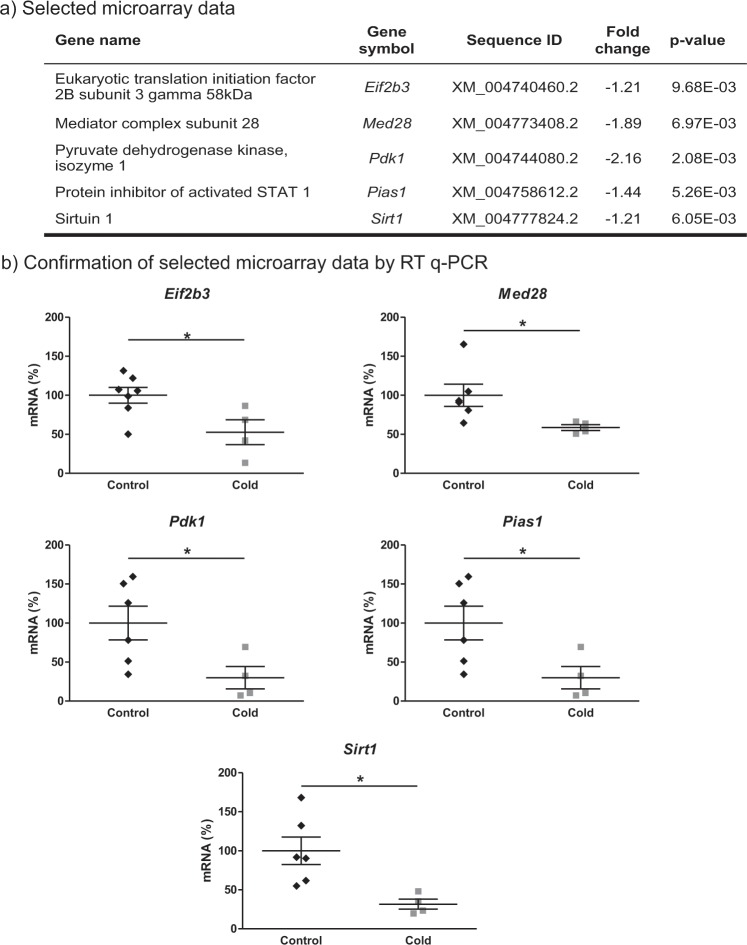
Microarray data confirmation. (**a**) Selected microarray data to be confirmed consisting of genes based on interesting pathways affected by cold exposure in PBMC *vs* their respective controls. Fold change indicates PBMC expression levels in cold-exposed animals over control group. (**b**) Microarray confirmation by RT-qPCR. Results are presented as dot plots (n = 4–7) of ratios of specific mRNA levels relative to *Apoo*, and are expressed as a percentage of the value of the control group that was set to 100%. Means ± S.E.M. are included. *indicates values significantly different *vs* control animals (Mann-Whitney U-test, p < 0.05).

In order to validate the microarray analysis, mRNA levels of five genes representative of the most regulated pathways (p-value < 0.01) (shown in Fig. 3a) were measured by real-time RT-qPCR in PBMC samples. As represented in Fig. 3b, the expression of a key regulator of protein synthesis, *Eif2b3*, and of the transcriptional co-activator, *Med28*, decreased in cold-exposed animals, as well as the expression of two energy metabolism key regulators, *Pdk1* and *Sirt1*; and the expression of *Pias1*, involved in lipogenesis, which was also decreased by cold exposure. These genes followed the same regulatory pattern as revealed in the microarray analysis (Fig. 3a).

**Figure 4:**
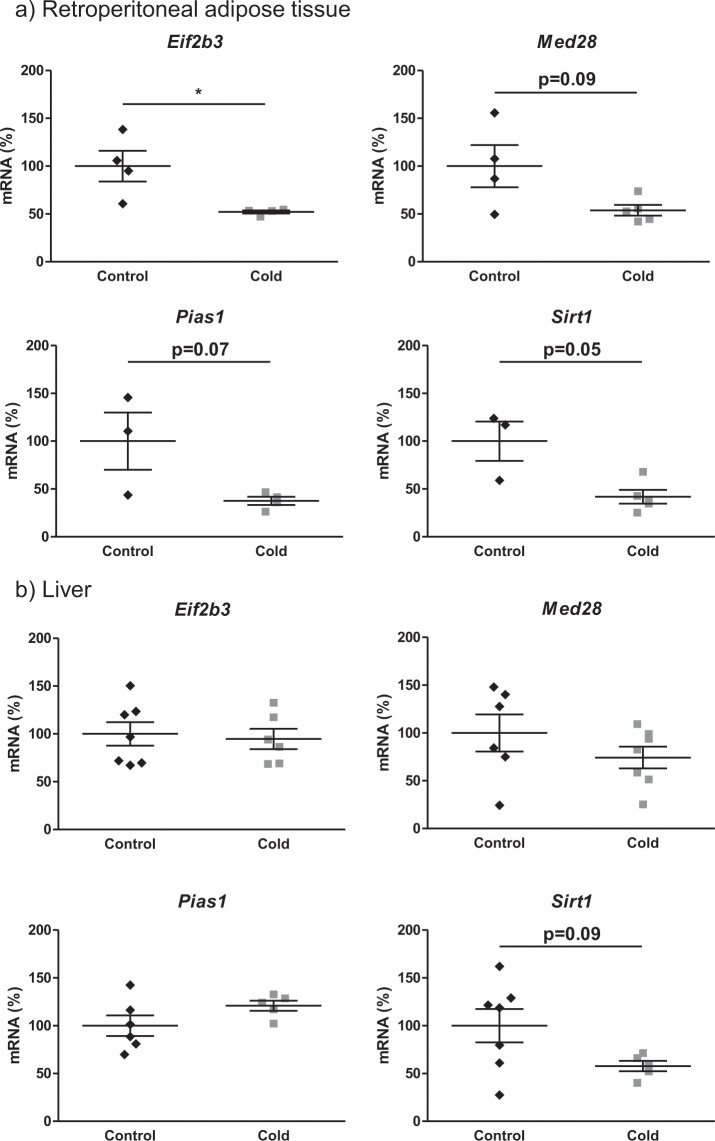
Retroperitoneal adipose tissue (**a**) and liver (**b**) mRNA expression analysis of selected genes. Results are presented as dot plots (n = 3–7) of ratios of specific mRNA levels relative to *Apoo*, and are expressed as a percentage of the value of the control group that was set to 100%. Means ± S.E.M. are included. *indicates values significantly different *vs* control animals (Mann-Whitney U-test, p < 0.05 or indicated when different).

Our group has repeatedly demonstrated that PBMC gene expression reflects the regulatory patterns observed in internal organs/tissues^20,21^. To see whether, in this case, our results were truly a reflection of the effect of cold exposure on relevant tissues, the expression of four relevant genes affected in the microarray analysis were analysed, in retroperitoneal white adipose tissue and in the liver. The genes selected were: *Eif2b3, Med28*, *Pias1*, and *Sirt1*. As observed in PBMC, we found decreased expression of the four genes analysed in the retroperitoneal white adipose tissue of cold-exposed animals (Fig. 4a), being significant for *Eif2b3* and showing a statistical trend (p < 0.1) for the other three: *Med28*, *Pias1* and *Sirt1* (Mann-Whitney *U* test). In liver, only a trend towards decreased expression in the cold group of the key energy metabolism regulator *Sirt1* was observed (Fig. 4b).

## Discussion

As far as we know this is the first study characterising the effect of cold on global gene expression in PBMC and how this is reflected in other tissues in ferrets. To date, most of the published data of cold effects on mammalian systems have emerged from studies aimed at specific processes, such as adaptive thermogenesis, mainly using rodents as animal model^11,22–25^. On the other hand, general cellular processes affected by cold exposure in mammals have been mainly evaluated in cell cultures or in hibernating mammals and mostly show a severe reduction in non-essential cellular processes to minimise catabolic pathways that utilise ATP^26,27^.

A global gene expression analysis was performed in PBMC from control and cold-exposed ferrets. Ferrets are an animal model closer to humans than the widely used rodents in several aspects, such as adipose tissue organisation and function, and immune response^14,15,28,29^. PBMC constitute an interesting tool to analyse the effects of cold on overall metabolism, as this cell fraction is known to express up to 80% of the human genome, mimicking gene expression response of internal organs in response to different stimuli^30^. In the present study, we show that gene expression analysis in PBMC is an interesting option to analyse the general effects of cold exposure on metabolism.

One-week 4 °C cold exposure had a severe impact on the PBMC transcriptome, especially affecting the expression of genes involved in gene expression regulation. Gene expression was, in fact, the most affected process, with 121 genes differentially expressed in cold *vs* control-exposed animals, most of which (101) were down-regulated. Previously, in different types of mammalian cells, inhibition of transcription and translation, inhibition of RNA degradation, and alternative splicing of pre-mRNA have been described as mechanisms explaining effects of cold exposure on gene expression^6^. Accordingly, our data reveal a clear inhibition of transcription factors, especially zinc finger proteins (e.g. *Zbed5, Zcchc11*, *Znf770*, *Zmym5*, *Zscan30*, and *Znf235*), as well as a down-regulation of genes involved in alternative splicing (such as *Ccdc12*, *Rbfox2*, *Rsrc1*, and *Syncrip*) and post-splicing regulators (*Upf3a* and *Upf3b*), which are essential for RNA maturation and post-transcriptional regulation. Moreover, our results show other affected genes related to gene expression regulation, among others, epigenetic regulators, interfering RNAs and coactivators (such as *Med28* and *Phf17*) and corepressors (such as *Bcor* and *Nab1*), which were mainly down-regulated and could contribute to the general gene expression inhibition observed in PBMC of cold-exposed ferrets.

The second most regulated process was the cell cycle, especially with genes involved in proliferation and cell-cycle regulation being down-regulated. Down-regulation of cell cycle as a consequence of cold exposure has been described for mammalian cultured cells and hibernating mammals^6,18,31^. Cold exposure was also observed to inhibit the expression of pro-apoptosis related genes (such as *Rnf122*) and increase the expression of anti-apoptosis genes (such as *Arl6ip6* and *Bcl2l15*) in ferret PBMC. This is in concordance with previous results revealing increased expression of the anti-apoptotic protein BCL2 in neurons from cold-exposed rats^32^. This is of interest, as our group has previously described that PBMC can also mimic changes in gene expression occurring in brain as a result of different nutritional stimuli^21,33^. Cold exposure has also been reported to induce ribosomal disaggregation in studies performed in brain and kidney of ground squirrel (*Spermophilus tridecemlineatus*)^34,35^, by inhibiting protein synthesis. In this sense, our results reveal transcriptional inhibition by cold exposure of genes related to ribosomal biogenesis, e.g. *Nsun4*, and mitochondrial ribosomal translation, e.g. *Mrps31* and *Mrps9*, suggesting ribosomal disaggregation related to protein synthesis inhibition in PBMC of ferrets as occurs in *in vitro* studies and in hibernating mammal models. Moreover, decreased expression in PBMC of genes coding for translation initiation factors, such as *Eif2b3* and *Mtif3* was also observed. Cold exposure thus mainly inhibits key effectors of gene expression and cell cycle progression, as well as protein synthesis, as has been observed for other tissues, suggesting this as a general response to cold and enforcing the potential sentinel function of PBMC.

PBMC are composed of lymphocytes and monocytes and, thus, play a key role in immune function. Cold exposure is known to exert a regulatory role on inflammation^4^ and, accordingly, immune response turned out to be the third most highly altered process (Fig. 2B) indicative of a modulatory effect of cold exposure on the expression of immune-related genes in PBMC, which is supported by our Metacore analysis (Fig. 2A). The relationship between cold exposure and immune response is somewhat controversial, with some authors revealing that a cold stimulus induces minimal or no immunological alterations in humans^36,37^, while others show immunosuppressive effects^38–42^. Since PBMC are cells of the immune system, our global gene expression analysis contributes to the knowledge of the effect of cold exposure on immunity. Further, by using ferret as the animal model, we have recently shown a specific down-regulatory effect of cold exposure on perivascular adipose tissue, which is reflected in PBMC^18^. This is of interest as inflammation in this adipose depot has been related to cardiovascular complications^43,44^. In the present study, 67 genes with a role in immune response were affected by cold exposure (p < 0.01, Students’ *t-*test); 28 of these were down-regulated and 39 up-regulated. Affected genes were involved mainly in immune system maturation/activation, but genes involved in antigen recognition and presentation, antigen degradation, and cytokine signalling were also affected. A more detailed classification of the up-regulated genes shows that they include genes coding for proteins with an inhibitory role in the immune response. For example, one of the top up-regulated genes was *Siglec8*, which codes for a transmembrane cell surface protein whose expression on leukocytes has an immune-inhibitory role^45^. Therefore, global gene expression data are indicative of a general immunosuppression effect observed at PBMC level. This is relevant, as an exacerbated immune response has been related to multiple pathologies^46,47^; thus, our results hint at a potential for clinical therapies based on the molecular effects induced by cold exposure. These results reinforce expression analysis in ferret PBMC as an option to test potential immunosuppressive effects of pharmacological therapies. One limitation of our study is that we did not measure serum cytokines, as there is a lack of ferret specific antibodies and reagents to detect these circulating inflammatory mediators.

Finally, another relevant process affected by cold exposure was energy metabolism, with 26 genes differentially expressed in PBMC of control and cold-exposed animals (p < 0.01, Students’ *t-*test). Among the affected genes, we found two master regulators of energy balance, *Pdk1* and *Sirt1*, which were down-regulated by cold exposure in ferret PBMC. *Pdk1* encodes a master kinase with a key role in energy homeostasis in liver^48^. Particularly, PDK phosphorylates and thereby inhibits pyruvate dehydrogenase complex activity, which is responsible for pyruvate conversion into acetyl-CoA, which can then enter the tricarboxylic acid (TCA) cycle for ATP synthesis, or be used for fatty acid synthesis^49^. The pyruvate dehydrogenase complex is active in most tissues in the fed state, and suppressing its activity by PDK is crucial to maintaining energy homeostasis under extreme nutritional conditions in mammals^49^. Thus, tentatively, PDK1 inhibition in PBMC by cold exposure could be indicative of increased pyruvate dehydrogenase activity. This would increase acetyl-CoA flux directed towards the TCA cycle – which can potentially feed electron transport complexes in order to sustain increased metabolic fluxes – and not towards fatty acid synthesis, coincident with the decreased expression observed of lipogenic genes (e.g. *Lpin1* and *Pias1*). This gene expression regulation observed in PBMC for lipogenic genes probably reflects decreased lipogenic capacity in key homeostatic tissues in response to cold exposure. This is in line with increased circulating NEFA levels in cold-exposed ferrets, indicative of fat mobilisation. However, we also observed decreased expression of some genes related to fatty acid catabolism, i.e. *Azgp1* and *Ehhadh* in PBMC after cold exposure. This effect could be related to lower *Sirt1* expression, as has also been described by other authors in brown and white adipose tissue depots of rodents exposed to 4 °C^50^. *Sirt1* mRNA codes for a NAD^+^-dependent deacetylase enzyme that regulates several metabolic processes, e.g. promoting fat mobilisation and reducing lipid synthesis in white adipose tissue^51^. Indeed, the decreased expression of *Sirt1* and *Pias2* in PBMC of ferrets reflected a similar trend in their retroperitoneal white adipose tissue, supporting their role as a sentinel tissue. One of the well-known effects of cold exposure is the induction of adaptive thermogenesis, consisting of heat production in brown adipose tissue to maintain body temperature at the expenses of lipid mobilisation^2,3^. Moreover, cold exposure also induces the appearance of brown-like adipocytes in white adipose tissue depots, the so called brite or beige adipocytes, in a process known as browning^52^. We have previously reported, using rodents, that PBMC do not express the gene coding for the protein responsible of brown adipose tissue thermogenesis, UCP1, but they do express other markers indicative of adaptive thermogenesis and white adipose tissue browning^53^. Interestingly, in the present study, cold exposure in ferrets induced the expression of *Pm20d1* in PBMC. This gene codes for a peptidase, which produces N-acyl amino acids that directly increase mitochondrial uncoupling, even in cells lacking UCP1^25,54^. This result is in line with metabolic adaptations to cold exposure and, to our knowledge, this is the first report suggesting that UCP1-independent thermogenesis is potentially being reflected in PBMC.

In conclusion, we provide first evidence of the usefulness of PBMC as easily obtainable material to obtain gene expression biomarkers to analyse the effect of cold exposure on overall metabolism. Our whole genome microarray reveals that cold exposure affects the ferret PBMC transcriptome which, at least to some extent, reflects the expected adaptations observed in a key homeostatic tissue, adipose tissue, in our own animal model, as well as adaptations described in other animal models or in cell cultures. That is, a widespread down-regulation of genes involved in gene expression, cell cycle, signal transduction and protein synthesis; moreover, key energy metabolism genes were also affected, reflecting the previously reported response in internal homeostatic tissues. Finally, cold exposure induced an immunosuppressive gene expression pattern in PBMC, which, together with increased energy dissipation, could be of therapeutic interest in terms of obesity and cardiovascular disease protection.

## Materials and Methods

### Animal procedure

The animal protocol followed in this study was reviewed and approved by the Bioethical Committee of the University of the Balearic Islands and guidelines for the use and care of laboratory animals were followed. Three month-old male ferrets (*Mustela Putorius Furo* from Cunipic, Lleida, Spain) were distributed into two groups (n = 7): a control group, acclimatised to room temperature (22 ± 2 °C), and a cold group, acclimatised to 4 °C for one week housed individually. All animals were exposed to a light/dark cycle of 12 h and had free access to water and diet (Gonzalo Zaragoza Manresa SL, Alicante, Spain). Animals were weighed before and after cold exposure. At the end of the experimental period, ferrets were anesthetised using 10 mg/kg of ketamine hydrochloride (Imalgène 1000, Merial Laboratorios SA, Lyon, France) and 80 mg/kg medetomidine (Domtor, Orion Pharma, Espoo, Finland). Arterial blood was collected from the left ventricle and animals died by exsanguination. Afterwards, liver and different visceral (aortic perivascular, retroperitoneal, mesenteric, and gonadal) and subcutaneous (interscapular and inguinal) adipose depots were rapidly removed. Adipose depots were weighed to detect changes in adiposity. Blood samples from the left ventricle were collected using heparin in NaCl (0.9%) as anticoagulant. For plasma collection, blood samples were stored for 1 h at 4 °C and centrifuged at 1000 g for 10 min at 4 °C.

### Quantification of circulating parameters

Circulating glucose was measured using an Accu-Chek Glucometer (Roche Diagnostics, Barcelona, Spain).

### PBMC isolation

PBMC were isolated from blood samples by Ficoll gradient separation according to the instructions of the manufacturer (GE Healthcare Bio Sciences, Barcelona, Spain), with some modifications^18^.

### Total RNA isolation

Total RNA from PBMC samples was extracted using Tripure Reagent (Roche Diagnostics, Barcelona, Spain) and then purified with E.Z.N.A. MicroElute RNA Clean Up (Omega Biotek, Vermont, USA), followed by precipitation with 3M sodium acetate and absolute ethanol. Moreover, total RNA from retroperitoneal adipose and liver samples was extracted using Tripure Reagent (Roche Diagnostics, Barcelona, Spain) and purified by precipitation with 3M sodium acetate and absolute ethanol. RNA yield was quantified using a NanoDrop ND 1000 spectrophotometer (NanoDrop Technologies, Wilmington, DE, USA). The integrity of PBMC samples was measured on an Agilent 2100 Bioanalyzer with RNA 6000 Nano chips (Agilent Technologies, South Queensferry, United Kingdom). The integrity of retroperitoneal adipose tissue and liver RNA samples was confirmed using agarose gel electrophoresis.

### Microarray processing and normalisation

We used an Agilent array, designed for our laboratory by the Príncipe Felipe Research Centre consisting of 45,328 sequences, which code for a total of 19,299 unique genes. PBMC RNA samples of ferrets were used for microarray processing (n = 6 *vs* 4, control and cold groups respectively). One array per sample was performed for each PBMC sample. For the cold group, samples from 4 out of 7 animals were used for the array due to difficulties to obtain PBMC in two of the animals. This did not affect the significance of the results either in the microarray analysis or in the confirmation by reverse transcription quantitative real-time polymerase chain reaction, as cold exposure had a deep and clear impact on the PBMC transcriptome. Microarray labelling and processing (hybridisation, washing, scanning and normalisation) was performed as previously described^18^ using a whole genome ferret-specific gene expression microarray designed by us and the Genomics and Translational Genetics Service of the Príncipe Felipe Research Centre (Valencia, Spain). The microarray was designed as a 2 × 400 k G4861A (AMADID-064079) Agilent array (Agilent Technologies, Inc., Santa Clara, CA, USA).

### Microarray data analysis

Statistical differences between the cold-exposed group *vs* the control group were assessed by Students’ *t-*test in GeneMaths XT 2.12; the generated p-values were used to obtain insight into significantly affected genes. Heatmaps of gene mean-centred expression data were obtained using GeneMaths XT 2.12 with hierarchical clustering based on samples (columns) and genes (rows). Fold change calculations (cold over control) were performed in Microsoft Excel. The most regulated genes (p-value < 0.01) were manually classified and sub-classified for biological information using available databases (Genecards, NCBI, WikiPathways, PubMed), focussing on key biological domains, such as molecular function and biological process. Analysis of process networks with a threshold for significance set at p < 0.01 was performed using Metacore (Thomson Reuters, New York, NY, USA). Gene symbols were used as unique identifier and since the ferret species is missing as input option, human species was selected. Microarray data have been deposited in NCBI Gene Expression Omnibus (GEO) under accession number GSE62352.

### Reverse transcription quantitative real-time polymerase chain reaction (RT-qPCR) analysis

To validate the microarray data analysis, RT-qPCR was used to measure mRNA expression levels in PBMC samples of both control and cold groups. Additionally, selected genes of interest were analysed in retroperitoneal adipose tissue and liver samples of the control (n = 4–7) and cold groups (n = 5–7). The following genes were analysed: (a) in PBMC: *Eif2b3*, *Med28*, *Pdk1*, *Pias1*, and *Sirt1*; (b) in retroperitoneal adipose tissue and in liver: *Eif2b3*, *Med28*, *Pias1*, and *Sirt1*. Using iScript cDNA synthesis kit (BIO-RAD, Madrid, Spain), 50 ng of total RNA from PBMC, retroperitoneal adipose tissue, and liver, was reverse transcribed to cDNA in an Applied Biosystems 2720 Thermal Cycler (Applied Biosystems, Madrid, Spain). Each PCR was performed from 1/5 diluted PBMC cDNA or 1/10 diluted adipose tissue or liver cDNA, forward and reverse primers (5 µM), and Power SYBER Green PCR Master Mix (Applied Biosystems) in a total volume of 11 µl, with the following profile: 10 min at 95 °C, followed by a total of 40 temperature cycles (15 s at 95 °C and 1 min at 60 °C for *Apoo* and 62 °C for the other genes) with a final cycle of 15 s at 95 °C, 1 min at 60 °C and 15 s at 95 °C. In order to verify the purity of the products, a melting curve was produced after each run according to the manufacturer’s instructions. Threshold cycle (Ct) was calculated using the instrument’s software (StepOne Software v2.0, Applied Biosystems) and the relative expression of each mRNA was calculated as a percentage of control animals, using the 2^−ΔΔCt^ method^55^. Data were normalised against the reference genes Apolipoprotein O (*Apoo*) for PBMC, adipose tissue, and liver. *Apoo* was selected as reference gene because it showed equal and high expression in the microarray for control and cold groups, with minimal variation over all samples. Primers for the different genes, described in Table 4, were obtained from Sigma Genosys (Sigma-Aldrich Química SA, Madrid, Spain).

**Table 4:** Nucleotide sequences of primers and amplicon size used for real-time RT-qPCR amplification.

Gene	Forward primer (5′-3′)	Reverse primer (5′-3′)	Amplicon size (bp)
*Eif2b3*	acgcaggttggagagaagtc	acgctgtcttggatgttgc	140
*Med28*	ggcttgctttgcttctcttg	acttgctctggtttctggaca	171
*Pdk1*	tcaccaggacagccaataca	ctcggtcactcatcttcacg	184
*Pias1*	tggagttgatggatgcttga	tggagatgcttgatgtggaa	223
*Sirt1*	ttcaccaccacattcttcagt	agccaacattcctcacatct	188
*Apoo* (Reference gene)	acgcaggttggagagaagtc	acgctgtcttggatgttgc	140

*Eif2b3*, Eukaryotic translation initiation factor 2B subunit 3 gamma 58KDa; *Med28*, Mediator complex subunit 28; *Pdk1*, Pyruvate dehydrogenase kinase isozyme 1; *Pias1*, Protein inhibitor of activated STAT1;* Sirt1*, Sirtuin 1; *Apoo*, Apolipoprotein O.

### Statistical analysis

Differences in real-time RT-qPCR data between control and cold groups were analysed using the Mann-Whitney *U* test. Analysis was performed with SPSS for Windows (version 15.0; SPSS, Chicago, IL, USA). Threshold of significance was defined at p-value < 0.05 and a trend at p < 0.1. The statistical analysis of the microarray data has been indicated above in the microarray data analysis section.

## Supplementary information

Table 2

Table 3

Supplementary Figures
