# Supplementary material for: Effects of cold exposure revealed by global transcriptomic analysis in ferret peripheral blood mononuclear cells
Source: Sci Rep. 2019 Dec 27;9:19985. doi: 10.1038/s41598-019-56354-6 (PMC6934835; doi:10.1038/s41598-019-56354-6)
Supplement: Supplementary file 3 — Supplementary Figures [file 41598_2019_56354_MOESM3_ESM.pdf]

Effects of cold exposure revealed by global transcriptomic analysis in ferret peripheral blood mononuclear cells

Bàrbara Reynés[1,2,3], Evert M. van Schothorst[4], Jaap Keijer[4], Andreu Palou[1,2,3]*, Paula Oliver[1,2,3]

[1]Laboratory of Molecular Biology, Nutrition and Biotechnology (Nutrigenomics and Obesity group), University of the Balearic Islands, Palma, Spain. [2]CIBER de Fisiopatología de la Obesidad y Nutrición (CIBEROBN), Madrid, Spain. [3]Balearic Islands Health Research Institute (IdISBa), Palma, Spain. [4]Human and Animal Physiology, Wageningen University, Wageningen, The Netherlands.

Supplementary Figure 1

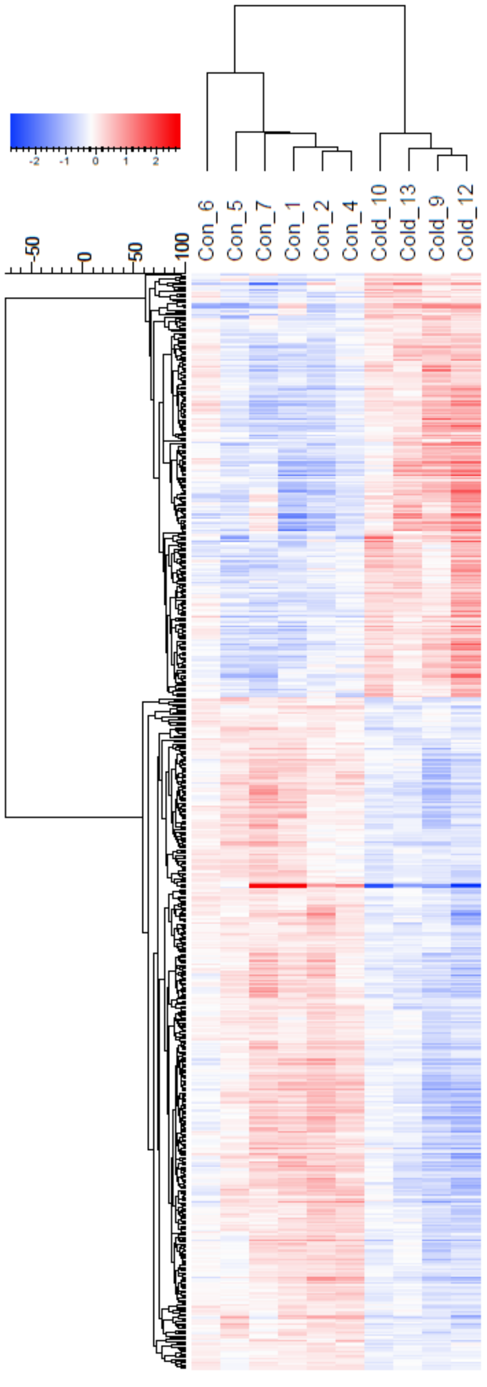

Heatmap showing differential expression for 611 genes by cold exposure in PBMC. Differential PBMC gene expression in cold *vs* control animals (p-value<0.01, Students' *t*-test, columns) is shown as mean-centred expression data per gene (row), with its associated Fold Change (log2 scale) of cold over control group by color-coding; scale bar is shown on the top.

Supplementary Figure 2

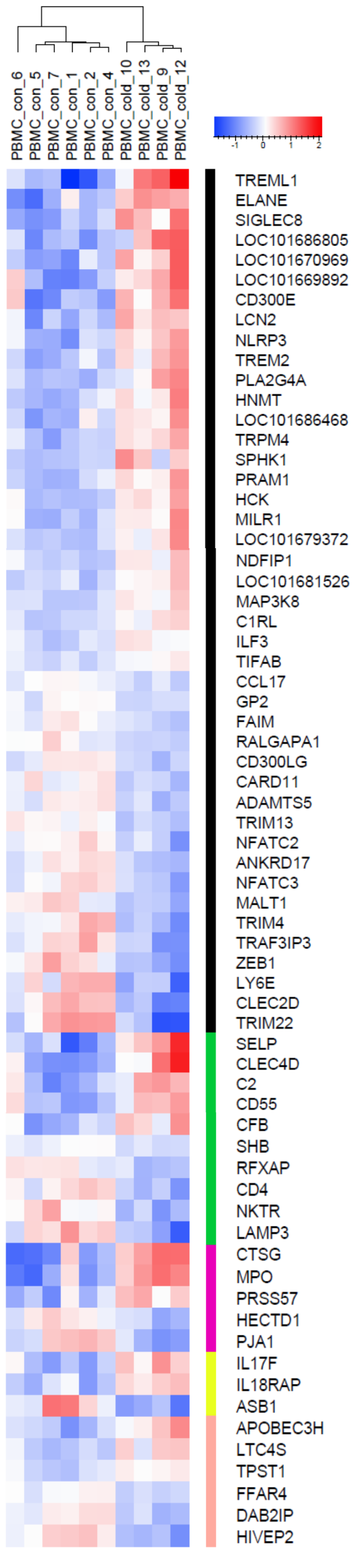

Heatmap showing differential expression of 67 immune response-related genes by cold exposure in PBMC. Differential PBMC gene expression in cold *vs* control animals ($p<0.01$, Students' *t*-test, columns) is shown as mean-centred expression data per gene (row), with its associated Fold Change (log2 scale) of cold over control group by color-coding; scale bar is shown on the top. Sub-classification of the genes is shown by coloured bars and their order within the subgroups is based on fold change. Black: immune system maturation/activation; green: antigen recognition/presentation; purple: antigen degradation; yellow: cytokine signalling; pink: others.

Supplementary Figure 3

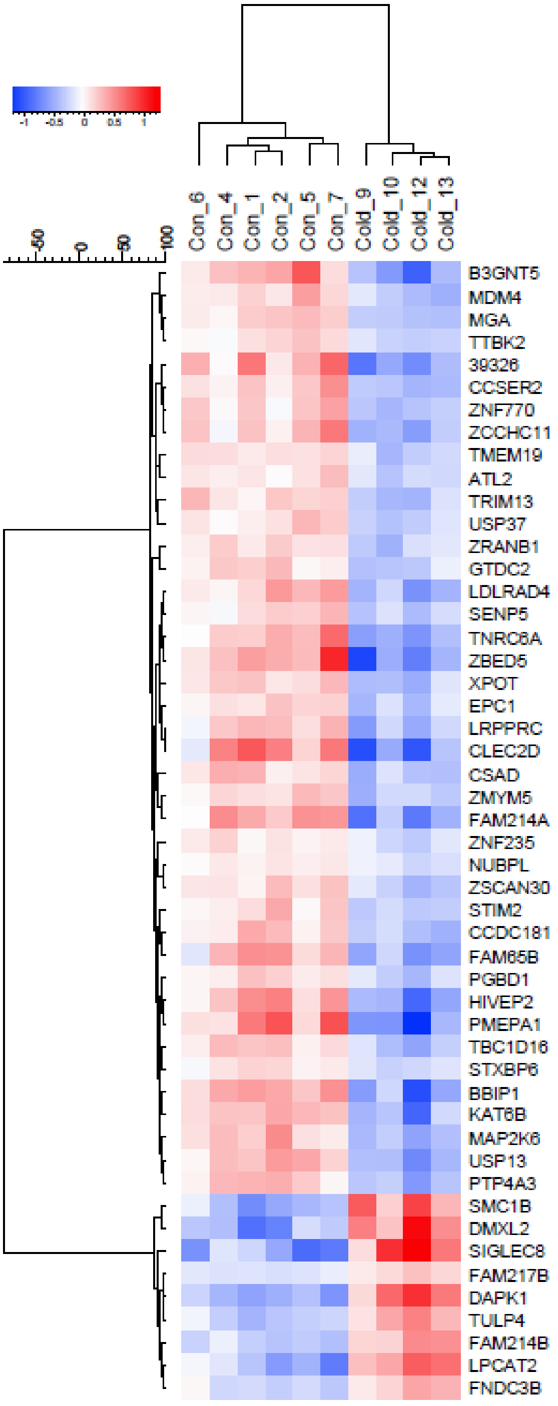

Heatmap showing gene expression of the top 50 regulated genes affected by cold exposure based on their p-value expressed in PBMC of cold *vs* control exposed ferrets. Differential PBMC gene expression in cold *vs* control animals (p-value<0.01, Students' *t*-test, columns) is shown as mean-centred expression data per gene (row), with its associated Fold Change (log2 scale) of cold over control group by color-coding; scale bar is shown on the top.
